# LncRNA LTSCCAT promotes tongue squamous cell carcinoma metastasis via targeting the miR-103a-2-5p/SMYD3/TWIST1 axis

**DOI:** 10.1038/s41419-021-03415-2

**Published:** 2021-02-01

**Authors:** Mo Liu, Qingwen Liu, Song Fan, Feng Su, Chun Jiang, Guanming Cai, Youyuan Wang, Guiqing Liao, Xinyuan Lei, Weixiong Chen, Junming Bi, Weiqi Cheng, LuoDan Zhao, Yi Ruan, Jinsong Li

**Affiliations:** 1 Guangdong Provincial Key Laboratory of Malignant Tumor Epigenetics and Gene Regulation of Sun Yat-Sen Memorial Hospital, Guangzhou, China; 2 Department of Periodontology, Sun Yat-Sen Memorial Hospital of Sun Yat-Sen University, Guangzhou, China; 3 Department of Oral and Maxillofacial Surgery, Sun Yat-Sen Memorial Hospital of Sun Yat-Sen University, Guangzhou, China; 4 Department of Urology, Shunde Hospital, Southern Medical University, Foshan City, Guangdong Province China; 5 Department of Urology, Sun Yat-Sen Memorial Hospital of Sun Yat-Sen University, Guangzhou, China; 6 Department of Oral and Maxillofacial Surgery, Guanghua School of Stomatology, Sun Yat-Sen University, Guangdong, China

**Keywords:** Oral cancer, Prognostic markers

## Abstract

Abnormal expression of long-noncoding RNA is involved in the tumorigenesis and progression of various cancers, but the potential molecular regulatory mechanisms are unclear. Microbial flora and chronic inflammation, such as periodontitis, which is associated with oral cancer, affect the occurrence and progression of tumors. Accordingly, we stimulated the tongue squamous cell carcinoma (TSCC) cell lines CAL27 and SCC15 with a low concentration of lipopolysaccharide (LPS) from *Porphyromonas gingivalis* (P.g) for 6 days and then performed LncRNA sequencing on P.g-LPS-treated CAL27 cells and untreated CAL27 cells. LTSCCAT was upregulated in P.g-LPS-treated CAL27 cells compared with untreated CAL27 cells. LTSCCAT induced epithelial–mesenchymal transition and promoted the invasion and metastasis of TSCC in vitro and in vivo. LncRNA LTSCCAT was upregulated in TSCC patients with periodontitis and was correlated with metastasis and poor prognosis. We predicted through an online database and confirmed by dual-luciferase reporter assays that LTSCCAT is a competitive endogenous RNA for the regulation of miR-103a-2-5p. Another dual-luciferase reporter assay confirmed that miR-103a-2-5p has a binding site at the 3′-UTR of the histone methylation transferase SMYD3 and inhibits its translation. Chromatin immunoprecipitation experiments demonstrated that SMYD3 binds directly to the promoter region of TWIST1 and promotes its transcription, which is related to H3K4 trimethylation. The effect of pcDNA/LTSCCAT on expression was attenuated by miR-103a-2-5p mimics. The RF and SVM classifier predicts that LTSCCAT can bind to SMYD3, whereas the RNA immunoprecipitation (RIP) assay confirms that it cannot. In addition, we predicted the combination of LTSCCAT and SMYD3 through software, but the RIP assay confirmed that LTSCCAT could not be combined with SMYD3. For the first time, we showed that periodontitis promotes the invasion and metastasis of TSCC and clarified the molecular mechanism of LTSCCAT to promote invasion and metastasis of TSCC, providing a potential therapeutic target for clinical treatment of TSCC.

## Introduction

Tongue squamous cell carcinoma (TSCC) is one of the most common malignant tumors in the oral cavity. As tongue tissue is rich in lymphatic vessels and blood circulation and has frequent movements, TSCC has a higher rate of local infiltration and a higher probability of cervical lymph node metastasis than any other oral cancer^1,2^. The prognosis of TSCC patients with cervical lymph node metastasis was significantly worse than that of patients without cervical lymph node metastasis. Cervical lymph node metastasis is an important factor leading to the death of TSCC patients. In recent years, abnormal expression of long-noncoding RNAs (LncRNAs) was shown to promote tumor invasion and metastasis.

LncRNAs, which are over 200 nt in length and do not encode proteins, were originally thought to be “noises” of genome transcription. Subsequently, many studies have reported that LncRNAs can regulate gene expression at the epigenetic, transcriptional, and post-transcriptional levels, which is closely related to the occurrence, development, and prevention of human diseases. In clinical treatment, the abnormal expression of LncRNAs in the cancer tissue of patients is related to different prognoses and is often associated with metastasis and poor prognosis. For example, LncRNA GMAN was upregulated in gastric cancer and associated with metastasis^3^. Similarly, the LncRNA LNMAT1 promoted the lymphatic metastasis of bladder cancer by recruiting macrophages^4^. A previous study indicated that LncRNA CILA1 promoted metastasis and chemoresistance of TSCC via the Wnt/β-catenin signaling pathway^5^. Although many studies have focused on tumor metastasis and abnormal expression of LncRNA, the molecular mechanisms remain poorly understood.

Microbial flora and chronic inflammation, such as periodontitis, have been shown to be associated with tumorigenesis and development^6–9^ as an independent risk factor for oral cancer^10–13^. Periodontitis is a common oral chronic inflammation caused by bacterial infection. *Porphyromonas gingivalis* (P.g), a major causative agent of periodontitis, has been shown to be present in cancer tissues at a higher level than normal tissues and is associated with subgingival plaques^14^. However, how periodontitis and its pathogens promote the invasion and metastasis of TSCC is not fully understood.

As a histone methylation transferase, SMYD3 is capable of dimethylating or trimethylating genomic proteins (such as H3K4, H3K27, H4K20) and has an important role in transcriptional regulation^15,16^. SMYD3 can accept the methyl group provided by S-adenosylmethionine and methylate the related histone H3 lysine 4 (H3K4), resulting in a spatial structural change in chromatin, which controls gene transcription^17,18^. SMYD3 can inhibit tumor cell apoptosis, promote cell proliferation^19^, invasion and migration^20–22^ and spread by affecting downstream oncogenes^15^, and cell cycle regulatory genes^23^.

In this study, LncRNA sequencing was performed on the TSCC cell lines, CAL27 and CAL27, treated with P.g-derived lipopolysaccharide (LPS) at low concentrations for 6 days. We demonstrated that a LncRNA, which we termed LPS-induced TSCC-associated transcript (LTSCCAT), was upregulated in P.g-LPS-treated CAL27, and its high expression was associated with periodontitis in patients with TSCC. In addition, we showed that LTSCCAT directly negatively regulates the expression of miR-103a-2-5p, which has binding sites on the 3′UTR of SMYD3 and inhibits its translation. Knockdown of LTSCCAT or overexpression of miR-103a-2-5p inhibited metastasis in TSCC cells. Meanwhile, histone methyltransferase SMYD3 binds directly to the promoter region of the transcription factor Twist1 and promotes transcription of Twist1, which is involved in the trimethylation of H3K4. Moreover, we predicted that LTSCCAT could bind to SMYD3, however the RIP assay confirms that it cannot. Our results demonstrated for the first time that LTSCCAT promotes TSCC metastasis by targeting the miR-103a-2-5p/SMYD3/TWIST1 axis, which may be the theoretical and therapeutic basis for periodontitis to promote the progression of TSCC.

## Results

### P.g-derived LPS induces EMT in TSCC cells

**Fig. 1:**
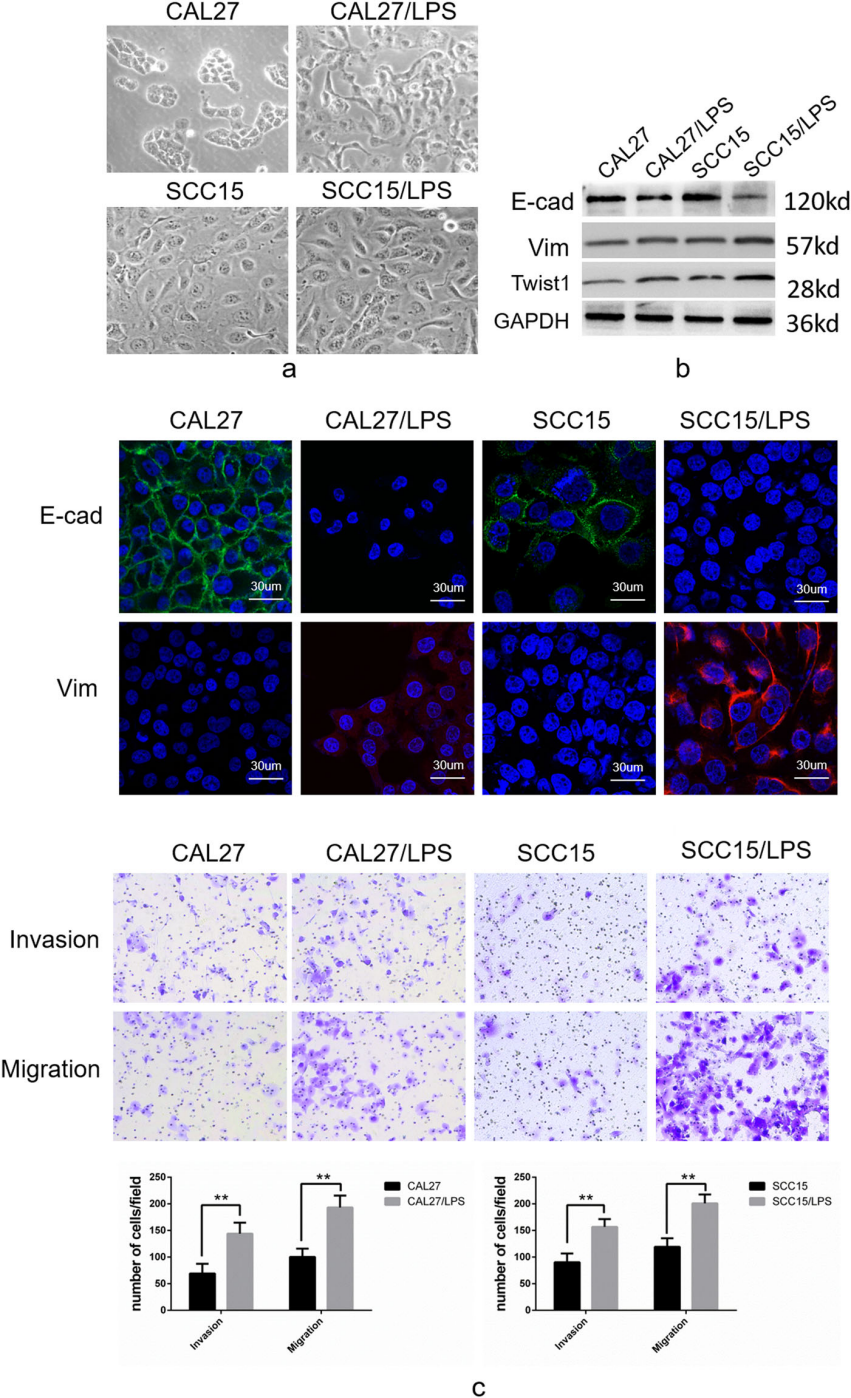
Epithelial–mesenchymal transition (EMT) was induced in tongue squamous carcinoma cell lines by LPS from *Porphyromonas gingivalis*. **a** The morphological changes in CAL27 and SCC15 cells after P.g-LPS induction from a tightly connected epithelial phenotype to an evacuated interstitial phenotype (×200). **b** Western blot analyses showed that E-cadherin protein expression was decreased in CAL27 and SCC15 cells induced by P.g-LPS, whereas vimentin and the EMT-related transcription factor Twist1 protein levels were increased. **c** Immunofluorescence also showed a decrease in the epithelial marker E-cadherin and an increase in the interstitial marker vimentin. **d** Transwell invasion and metastasis assays confirmed that P.g-LPS-induced CAL27 and SCC15 cells showed higher invasion and metastasis than the control cells. Error bars represent the mean±S.D from three independent experiments. (**P < 0.01).

After 6 days of induction of the TSCC cells CAL27 and SCC15 with 20 µg/ml LPS derived from P.g, we observed that P.g-LPS-treated CAL27 and SCC15 showed epithelial–mesenchymal transition (EMT) compared with their parent cell lines (Fig. 1a). Western blot and immunofluorescence experiments demonstrated that E-cadherin was downregulated and vimentin was upregulated (Fig. 1b, c). Cell invasion and migration were significantly enhanced (Fig. 1d). In addition, we found that the expression of Twist1, a tumor metastasis-associated transcription factor, was upregulated in P.g-LPS-treated CAL27 and SCC15 cells (Fig. 1b).

### LncRNA LTSCCAT is upregulated in TSCC patients with periodontitis and is correlated with metastasis and poor prognosis

**Fig. 2:**
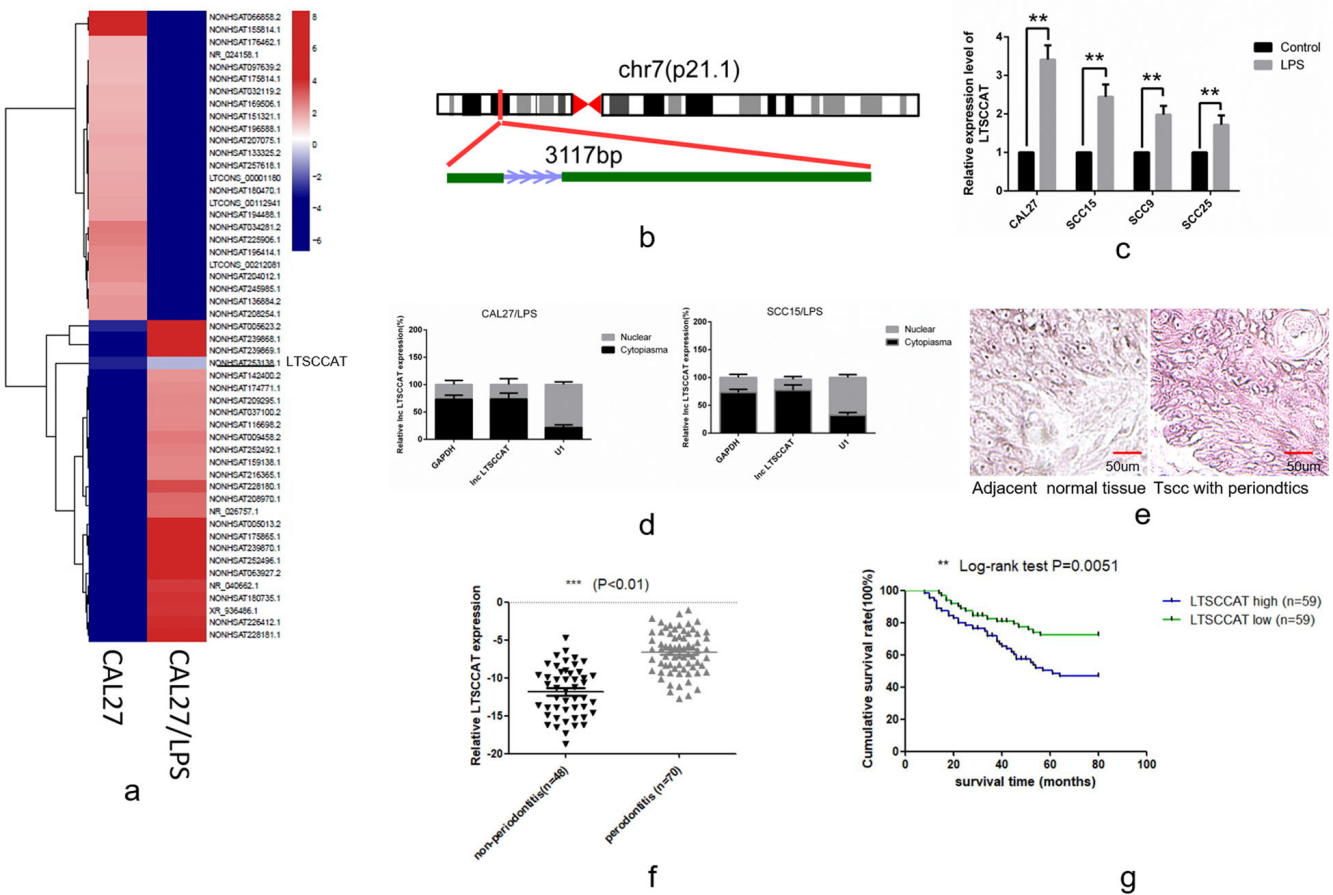
Expression and clinical value of LTSCCAT in tongue squamous cell carcinoma. **a** Heat maps were generated based on the sequencing results, showing the expression levels of LncRNA in CAL27 and CAL27/LPS cells (unsupervised cluster analysis). Each row represents one LncRNA, the left column represents the expression of CAL27 cells, and the right column represents the expression of P.g-LPS-induced CAL27 cells. Red represents a high expression level, and blue represents a low expression level. **b** According to the sequencing results of LncRNAs, LTSCCAT located 113 bp downstream of the Twist1 gene has a homeostatic effect on Twist1 expression. **c** The sequencing results were verified in tongue squamous carcinoma cell lines and showed that P.g-LPS significantly upregulated the expression of LTSCCAT in CAL27, SCC15, SCC9, and SCC25 cells. Error bars represent the mean±S.D. from three independent experiments. (**P < 0.01). **d** Nuclear pulp separation experiments showed that LTSCCAT was predominantly expressed in the cytosol. **e** Representative images of in situ hybridization for LTSCCAT in TSCC with periodontitis tissues and non-periodontitis tissues (scale bar: 50 µm). **f** The expression of LTSCCAT in clinical specimens was detected by RT-QPCR, indicating that LTSCCAT expression is higher in patients with periodontitis than in those without periodontitis. (**P < 0.01). **g** Kaplan–Meier analysis revealed that high expression of LTSCCAT is associated with poor prognosis. The *p* values were assessed by the log-rank test. (**P < 0.01).

We performed LncRNA sequencing on P.g-LPS-treated CAL27 cells and untreated CAL27 cells and detected aberrantly expressed lncRNAs between them. According to the sequencing results, a total of 2261 LncRNAs were upregulated. LTSCCAT was one of the upregulated LncRNAs in P.g-LPS-treated CAL27 and is located on chromosome 7, ~113 bp downstream of Twist1 (Fig. 2a, b). We verified the expression of LncRNAs upregulated in the heat map in CAL27, SCC9, SCC15, and SCC25 cells, and only LTSCCAT was upregulated in all four cells. Therefore, we selected LTSCCAT for further research. Based on the 3′ RACE and 5′ RACE experiments, we obtained the full-length LTSCCAT, which is 3117 bp (Fig. 2b). Using RT-qPCR, we further validated the high expression levels of LTSCCAT in P.g-LPS-treated CAL27, SCC15, SCC9, and SCC25 cells compared with those of their untreated control cells (Fig. 2c). Using nucleoplasm separation experiments and in situ hybridization (ISH), we found that LTSCCAT was mainly expressed in the cytoplasm (Fig. 2d, e).

Then, we examined the expression levels of LTSCCAT in 118 TSCC tissues, which were divided into either the periodontitis group (*N* = 70) or the no periodontitis group (*N* = 48). LTSCCAT expression in tissues from TSCC patients with periodontitis was higher than that in TSCC patients without periodontitis (Fig. 2f). As expected, LTSCCAT had clinical significance and was related to tumor stage, lymph node metastasis and the presence or absence of periodontitis (Table 1). Moreover, Kaplan–Meier survival analysis showed that upregulation of LTSCCAT expression in TSCC tissues was significantly associated with a decrease in overall survival (Fig. 2g). These results indicated that LncRNA LTSCCAT promotes the invasion and metastasis of TSCC and may be a potential factor for predicting metastasis and prognosis in patients with TSCC.

**Table 1:** Relationship between the expression of LTSCCAT and SMYD3 and tumor stage, lymph node metastasis, and periodontitis.

Characteristic	LTSCCAT (%)		*P*	SMYD3 (%)		*P*
	No. of high expression	No. of low expression		No. of high expression	No. of low expression	
*Sex*			0.446			0.703
Male	39 (52.7)	35 (47.3)		36 (48.6)	38 (51.4)	
Female	20 (45.5)	24 (54.5)		23 (52.3)	21 (47.7)	
*Age*			0.851			0.573
<50	24 (51.1)	23 (48.9)		22 (46.8)	25 (53.2)	
*Node metastasis*			0.002*			0.016*
N0	18 (34)	35 (66)		20 (37.7)	33 (62.3)	
N^+^	41 (63.1)	24 (36.9)		39 (60)	26 (40)	
*Clinical stage*			0.003*			0.002*
III	20 (35.7)	36 (64.3)		18 (34)	38 (66)	
IV	39 (62.9)	23 (37.1)		41 (63.1)	21 (36.9)	
*Status*			0.002*			0.000*
Survival	23 (36.5)	40 (63.5)		19 (30.2)	44 (69.8)	
Death	36 (65.5)	19 (34.5)		40 (72.7)	15 (27.3)	
*Periodontitis*			0.000*			0.003*
Yes	46 (65.7)	24 (34.3)		43 (61.4)	27 (38.6)	
No	13 (27.1)	35 (72.9)		16 (33.3)	32 (66.7)	

Chi-square test. *p < 0.5.

### LncRNA LTSCCAT promotes EMT of TSCC in vitro

**Fig. 3:**
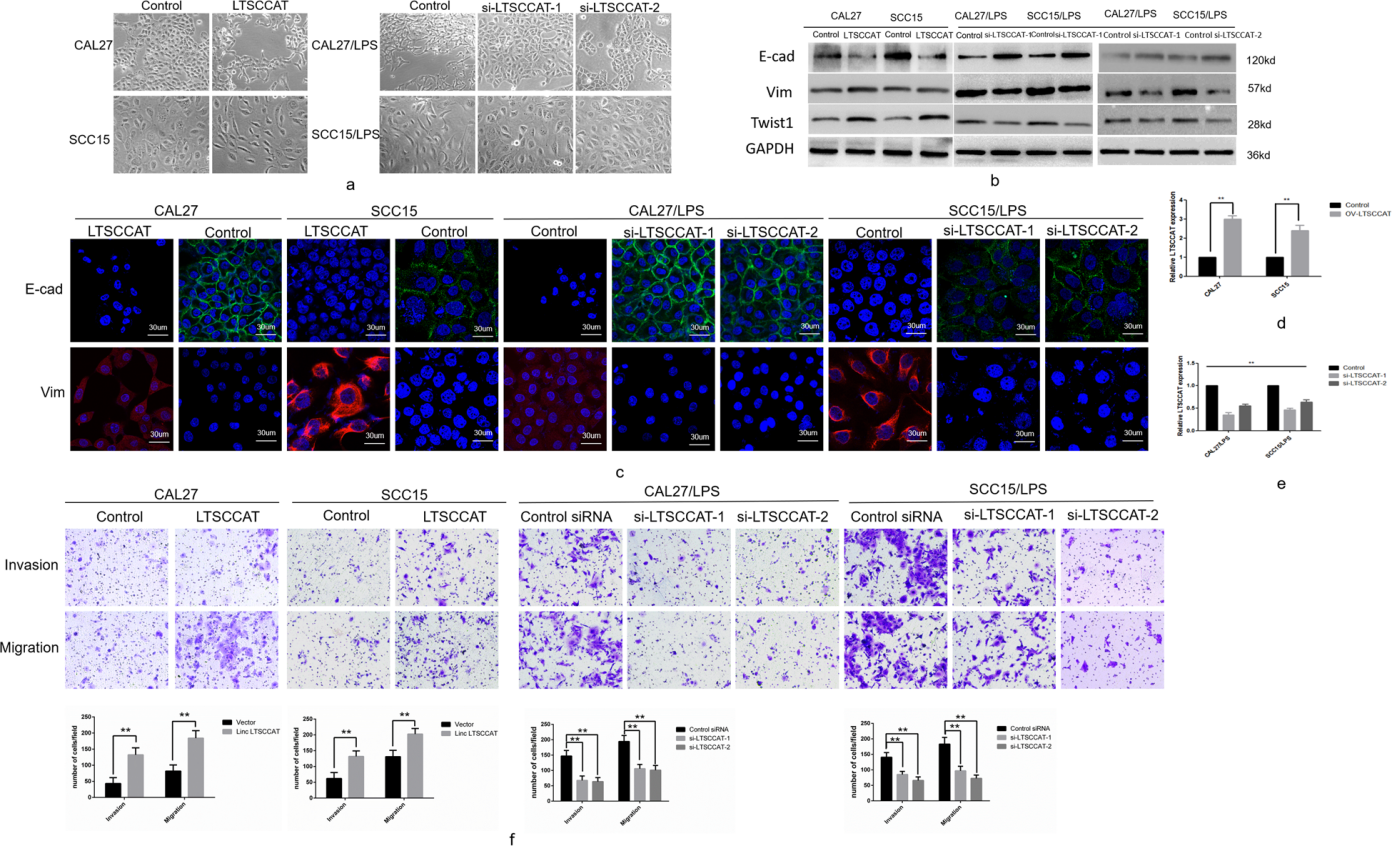
LTSCCAT promotes invasion and migration of tongue squamous cell carcinoma. **a** Left: after upregulation of LTSCCAT in CAL27 and SCC15 cells, cells transitioned from an epithelial morphology to interstitial morphology. Right: after knockdown of LTSCCAT expression with two siRNAs in CAL27/LPS and SCC15/LPS, cells gradually transitioned from an interstitial morphology to epithelial morphology. (×200). **b** Western blot analyses showed that after overexpression of LTSCCAT in CAL27 and SCC15 cells, E-cadherin decreased and vimentin and Twist1 increased. After knockdown of LTSCCAT with two siRNAs in CAL27/LPS and SCC15/LPS cells, E-cadherin increased and vimentin and Twist1 decreased. **c** Immunofluorescence results showed that E-cadherin was downregulated and vimentin was upregulated in CAL27 and SCC15 cells after overexpression of LTSCCAT, whereas E-cadherin was upregulated and vimentin was downregulated after knockdown of LTSCCAT with two siRNAs in CAL27/LPS and SCC15/LPS cells. **d** After overexpression of LTSCCAT in CAL27 and SCC15, the expression level of LTSCCAT was upregulated compared with control cells. **e** After knockdown of LTSCCAT with two siRNAs in CAL27/LPS and SCC15/LPS cells, the expression level of LTSCCAT was downregulated compared with control cells. **f** Transwell invasion and migration assays demonstrated that the invasive and migratory ability of CAL27 and SCC15 cells was increased after overexpression of LTSCCAT, and the invasion and migration of CAL27/LPS and SCC15/LPS cells decreased after LTSCCAT knockdown. Error bars represent the mean±S.D. from three independent experiments. (**P < 0.01).

We investigated the effects of LTSCCAT overexpression in CAL27 and SCC15 cells by transfection plasmids and detected EMT (Fig. 3a). The expression level of LTSCCAT after transfection of plasmid was higher than that of control group (Fig. 3d). Western blot and immunofluorescence experiments demonstrated that E-cadherin was downregulated and vimentin was upregulated (Fig. 3b, c). Twist1 was also upregulated (Fig. 3b). Transwell invasion and migration assays showed that LTSCCAT-overexpressing TSCC cells displayed increased invasion and migration (Fig. 3f).

We observed mesenchymal-epithelial transition (MET) in P.g-LPS-treated CAL27 and SCC15 cells after transfection with two LTSCCAT-targeted siRNAs compared with transfection with the negative control siRNA (Fig. 3a). The expression level of LTSCCAT after transfection of two siRNAs was lower than that of the control group (Fig. 3e). Western blot and immunofluorescence experiments showed upregulation of E-cadherin and downregulation of vimentin and Twist1 (Fig. 3b, c). Transwell invasion and migration assays revealed that knockdown of LTSCCAT in TSCC cells repressed the invasive and migratory abilities (Fig. 3f).

### SMYD3 promotes EMT of TSCC in vitro

**Fig. 4:**
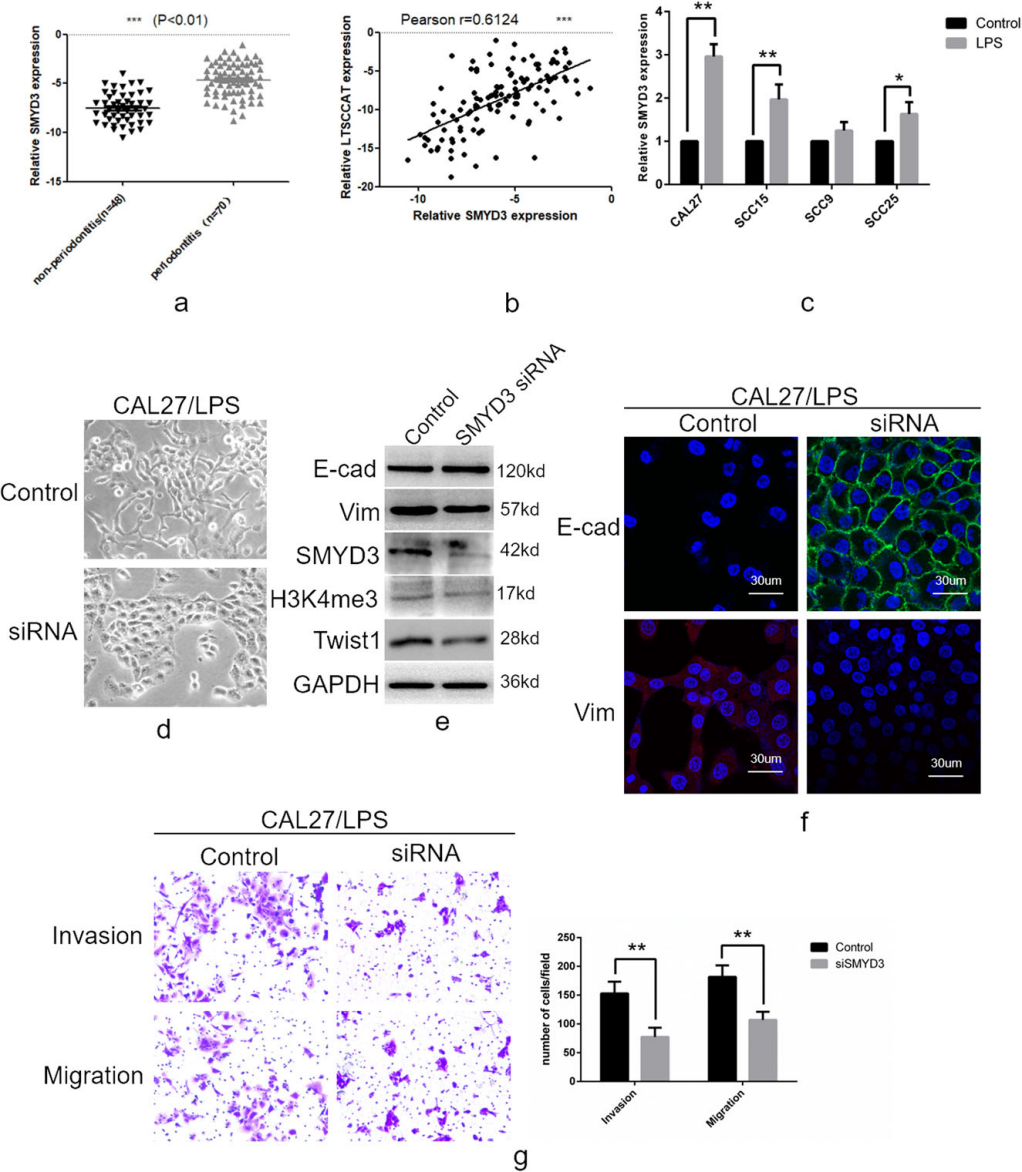
SMYD3 promotes epithelial–mesenchymal transition in tongue squamous cell carcinoma. **a** The expression of SMYD3 in clinical specimens was detected by RT-QPCR, indicating that SMYD3 expression in TSCC tissues is higher in patients with periodontitis than in those without periodontitis. (**P < 0.01). **b** Correlation analysis between LTSCCAT and SMYD3 was performed in TSCC tissues. (**P < 0.01). **c** The expression of SMYD3 was reduced in the P.g-LPS-induced TSCC cell lines CAL27, SCC15, and SCC25. **d** SiRNA knockdown of SMYD3 expression in CAL27/LPS cells resulted in a cell morphology transition from an interstitial phenotype to an epithelial phenotype. (×200). **e** Western blot analyses showed that E-cadherin was upregulated after knockdown of SMYD3, while vimentin, SMYD3, H3K4me3, and Twist1 protein levels were downregulated. **f** Immunofluorescence experiments showed that E-cadherin increased and vimentin was decreased after knockdown of SMYD3. **g** Transwell invasion and migration assays indicated that the invasion and migration ability of CAL27/LPS was decreased after siRNA knockdown of SMYD3. Error bars represent the mean±S.D. from three independent experiments. (**P < 0.01).

A recent report showed that SMYD3 can promote EMT in breast cancer^24^. We investigated whether SMYD3 also promotes EMT in TSCC, which may be related to the mechanism by which LTSCCAT promotes EMT. Reverse transcription-polymerase chain reaction (RT-PCR) showed that the SMYD3 expression in tissues from TSCC patients with periodontitis was higher than that in TSCC patients without periodontitis (Fig. 4a). Furthermore, LTSCCAT and SMYD3 expression showed a significant positive correlation in TSCC patients by Spearman’s correlation analysis (Fig. 4b). SMYD3 showed clinical significance and was related to tumor stage, lymph node metastasis, and the presence or absence of periodontitis (Table 1). We tested the expression levels of SMYD3 in P.g-LPS-treated CAL27, SCC15, SCC9, and SCC25 cells and their untreated control cells and found that SMYD3 was upregulated in CAL27, SCC15, and SCC25 cells (Fig. 4c).

After transfection of SMYD3-targeting siRNA and negative control siRNA into P.g-LPS-treated CAL27 cells, we observed MET. (Fig. 4d). The expression level of E-cadherin was upregulated and that of vimentin was downregulated; moreover, the expression of Twist1 and H3K4me3 decreased (Fig. 4e, f). Transwell invasion and migration assays revealed that knockdown of SMYD3 in CAL27/LPS cells repressed the invasive and migratory abilities (Fig. 4g).

### LTSCCAT acts as a competing endogenous RNA (ceRNA) by sponging miR-103a-2-5p and indirectly regulates the SMYD3/Twist1 pathway

**Fig. 5:**
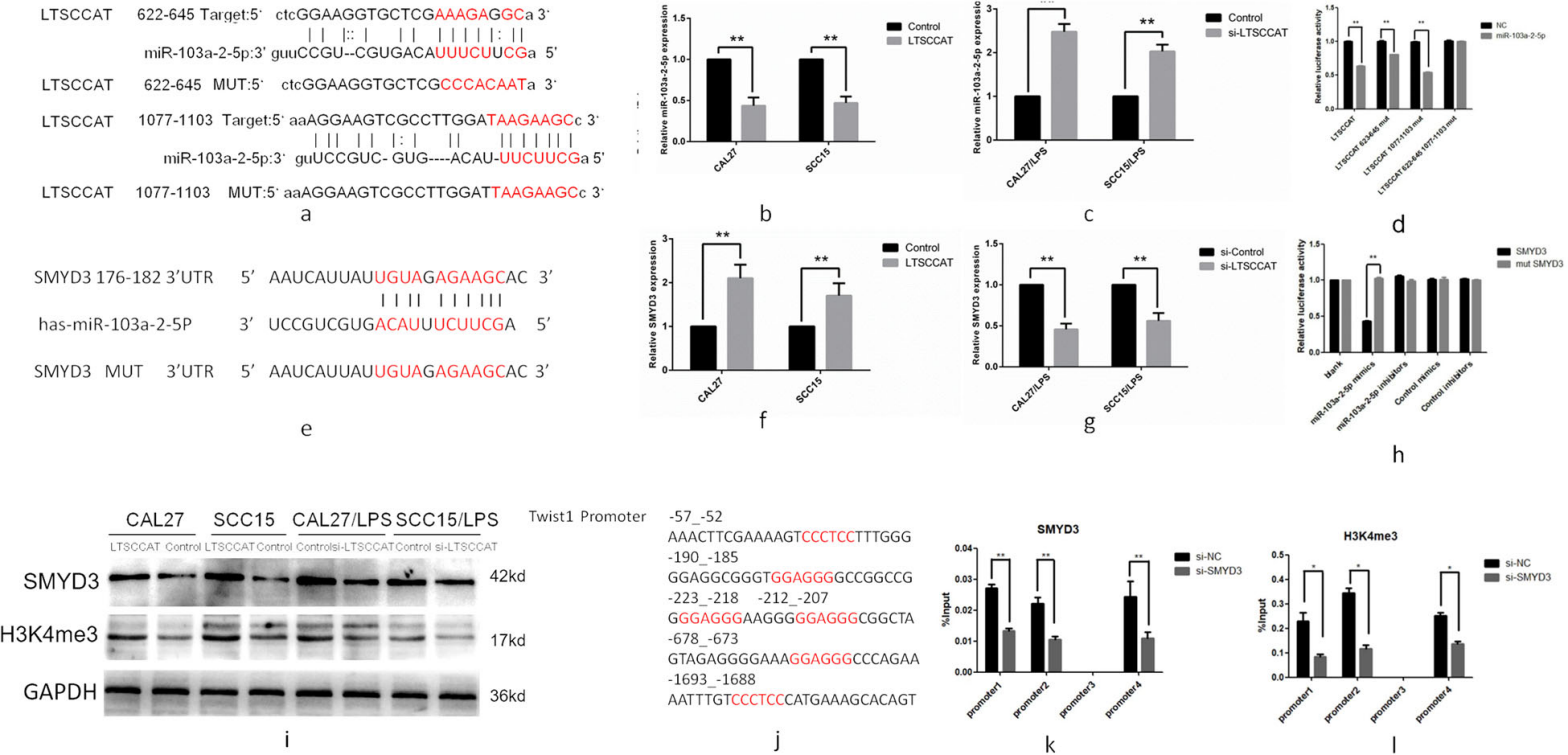
LTSCCAT promotes invasion and metastasis of TSCC via the miR-103a-2-5p/SMYD3/Twist1 signaling pathway. **a** Target sequence of LTSCCAT in the miR-103a-2-5p 3′-UTR predicted by RegRNA 2.0 as well as the mutated sequence. **b** Expression of miR-103a-2-5p was decreased in CAL27 and SCC15 cells overexpressing LTSCCAT. Error bars represent the mean±S.D. from three independent experiments. (**P < 0.01). **c** miR-103a-2-5p was downregulated in CAL27/LPS and SCC15/LPS cells after knockdown of LTSCCAT. Error bars represent the mean±S.D. from three independent experiments. (**P < 0.01). **d** Dual-luciferase reporter assays were performed by cotransfecting miR-103a-2-5p mimics with wild-type and mutant LTSCCAT in 293 T cells, which indicated that miR-103a-2-5p mimics could impair wild-type LTSCCAT promoter transcriptional activity compared with the negative control vector. However, no changes were observed in the mutation group. Error bars represent the mean±S.D. from three independent experiments. (*P < 0.05, **P < 0.01). **e** Target sequence of SMYD3 in the miR-103a-2-5p 3′-UTR, as well as the mutated sequence, was predicted by RegRNA 2.0. **f** SMYD3 was upregulated in CAL27 and SCC15 cells overexpressing LTSCCAT. Error bars represent the mean±S.D. from three independent experiments. (**P < 0.01). **g** SMYD3 was downregulated after knockdown of LTSCCAT in CAL27/LPS and SCC15/LPS cells. Error bars represent the mean±S.D. from three independent experiments. (**P < 0.01). **h** Western blot analysis showed that SMYD3 and H3K4me3 were upregulated after overexpression of LTSCCAT in CAL27 and SCC15 cells, and SMYD3 and H3K4me3 were downregulated after knockdown of LTSCCAT in CAL27/LPS and SCC15/LPS cells. **i** Wild-type SMYD3 and mutant SMYD3 were cloned into the luciferase plasmid and cotransfected with mimics and inhibitors of miR-103a-2-5p. The R/F ratio of miR-103a-2-5p mimics cotransfected with wild-type SMYD3 was significantly lower than that of the blank group and the mutant group, indicating that miR-103a-2-5p can interact with wild-type SMYD3. Error bars represent the mean±S.D. from three independent experiments. (**P < 0.01). **j** The Twist1 promoter contains four latent binding sites with SMYD3. **k**, **l** ChIP assays were performed with the SMYD3 antibody and H3K4me3 antibody, respectively, and purified DNA was analyzed by QPCR. The results confirmed that SMYD3 binds directly to Twist1 and promotes its transcription, which is associated with elevated levels of trimethylation of H3K4. Error bars represent the mean±S.D. from three independent experiments. (*P < 0.05, **P < 0.01).

Many studies have shown that LncRNAs can act as ceRNAs to bind to target miRNAs and inhibit their expression^25–27^. Bioinformatics prediction and analysis by RegRNA 2.0 (regrna2.mbc.nctu.edu.tw) revealed the presence of a binding site for hsa-miR-103a-2-5p in the LncRNA LTSCCAT sequence^28^ (Fig. 5a).

We investigated the consequences of LTSCCAT overexpression or knockdown in CAL27 and SCC15 cells by transfection plasmids or siRNAs and detected the hsa-miR-103a-2-5p levels. We found that LTSCCAT plasmid transfection downregulated the expression of hsa-miR-103a-2-5p, and LTSCCAT siRNAs upregulated the expression of hsa-miR-103a-2-5p (Fig. 5b, c). To further confirm the predictions of bioinformatics analysis, we performed a dual-luciferase reporter gene experiment. According to bioinformatics predictions, LTSCCAT may bind to miR-103a-2-5p at the 622–645 and 1077–1103 sites of its sequence (Fig. 5a). Therefore, we designed three mutants, including LTSCCAT mutated at 622–645 or 1077–1103 or simultaneously mutated at both 622–645 and 1077–1103, and cloned the three mutants and wild-type LTSCCAT into the luciferase plasmid. Next, we cotransfected these plasmids with miR-103a-2-5p into HEK293T cells and performed luciferase experiments to determine whether miR-103a-2-5p binds to LTSCCAT. Cotransfection of wild-type LTSCCAT with miR-103a-2-5p substantially inhibited luciferase activity, whereas cotransfection of mutant LTSCCAT with miR-103a-2-5p did not significantly inhibit luciferase activity. These results confirmed that the LTSCCAT sequence has two sites that bind to miR-103a-2-5p (Fig. 5d).

Furthermore, we wondered whether miR-103a-2-5p could target SMYD3 to inhibit EMT in TSCC cells. We found through bioinformatics analysis that miR-103a-2-5p has a binding site to the 3′-UTR of the histone methylation transferase SMYD3 mRNA (Fig. 5e). We investigated the consequences of overexpressing LTSCCAT in CAL27 and SCC15 cells or knockdown of LTSCCAT in CAL27/LPS and SCC15/LPS cells by transfection plasmids or siRNAs and detected the mRNA levels of SMYD3. We found that LTSCCAT plasmid transfection upregulated the expression of SMYD3 and LTSCCAT siRNAs downregulated the expression of SMYD3 (Fig. 5f, g). Western blot analysis showed that overexpressing LTSCCAT in CAL27 and SCC15 cells resulted in the upregulation of SMYD3 and H3K4me3. LTSCCAT knockdown in CAL27/LPS and SCC15/LPS cells resulted in the downregulation of SMYD3 and H3K4me3 (Fig. 5h). Next, we confirmed that miR-103a-2-5p can target SMYD3 mRNA by luciferase reporter gene assays (Fig. 5i).

According to previous reports, MYND, the amino acids at position 1–28 of SMYD3, can specifically bind to 5c-CCCTCC-3c or 5c-GGAGGG-3c in the promoter region of the relevant gene, allowing the SET domain to exert its methylation function^19^. We found that there are six sites of 5c-CCCTCC-3c or 5c-GGAGGG-3c in the promoter region of Twits1, which may directly bind to SMYD3 (Fig. 5j). Therefore, we further investigated whether SMYD3 regulates the expression of Twist1 at the transcriptional level by directly binding to the Twist1 promoter region through a chromatin immunoprecipitation (ChIP) assay. Based on the site in the literature where the promoter region may bind to SMYD3, we designed four primers for the Twist1 promoter region (Supplementary Table S1). Next, we transfected SMYD3 siRNAs and negative control siRNAs into P.g-LPS-treated CAL27 cells, and ChIP-qPCR confirmed that SMYD3 has three binding sites in the promoter region of Twist1. After SMYD3 knockdown, Twist1 promoter expression was decreased (Fig. 5k). Similarly, we performed ChIP assays in CAL27/LPS and CAL27/LPS SMYD3 knockdown cells with H3K4me3 antibody. The results showed that trimethylation of H3K4 is involved in the promotion of Twist1 transcription by SMYD3 (Fig. 5l).

We predicted the interaction probabilities of LTSCCAT and SMYD3 using the RF classifier and SVW classifier. The results showed that LTSCCAT probably directly interacts with SMYD3 (Fig. S1A). However, RNA immunoprecipitation (RIP) assays revealed that LTSCCAT could not interact with SMYD3 in TSCC cells (Fig. S1B), indicating that LTSCCAT cannot directly bind to SMYD3.

### miR-103a-2-5p inhibits TSCC metastasis by targeting SMYD3

**Fig. 6:**
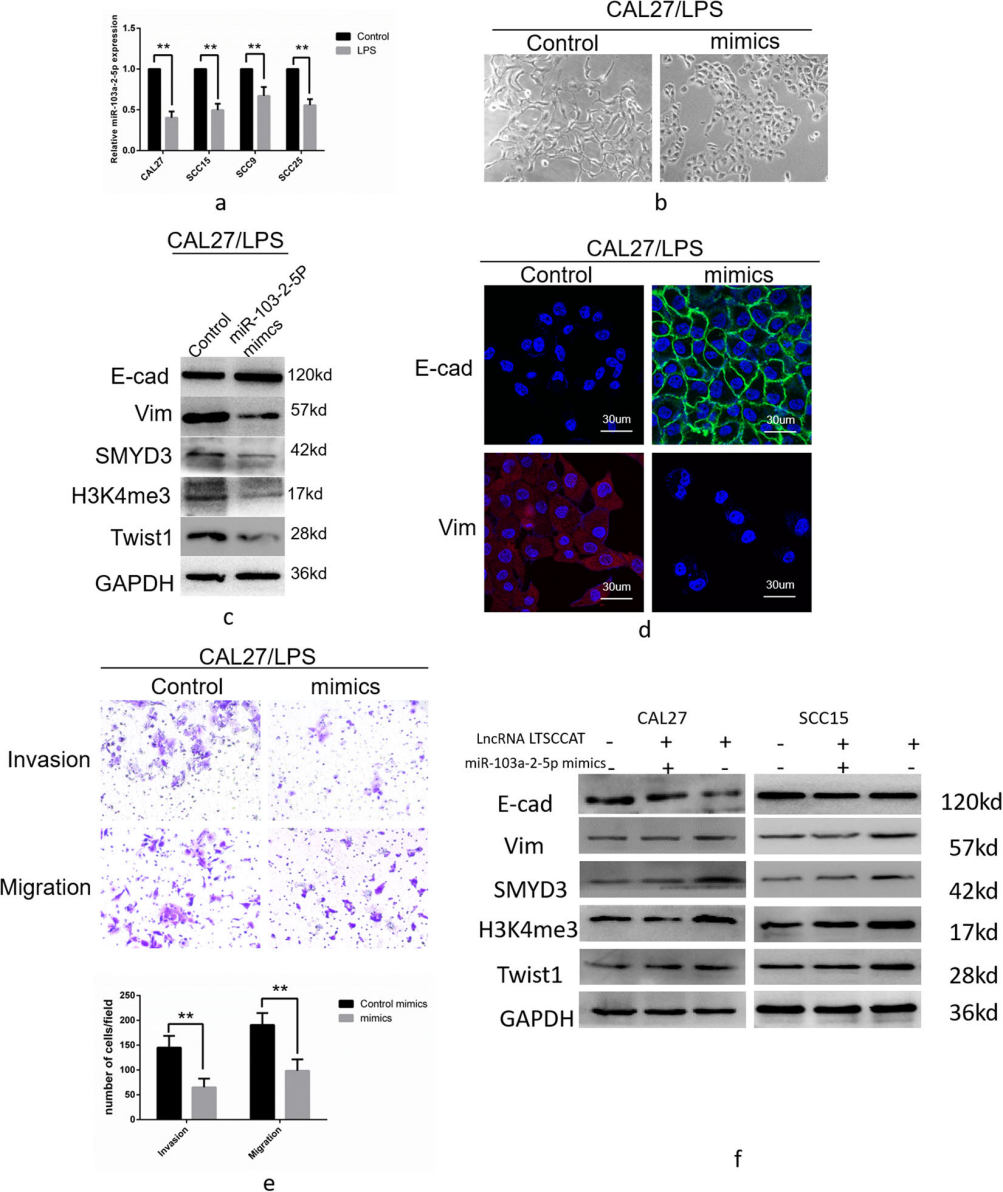
Overexpression of miR-103a-2-5p in CAL27/LPS cells inhibited the invasion and migration of tongue squamous cell carcinoma cells. **a** The expression of miR-103a-2-5p was reduced in the P.g-LPS-induced TSCC cell lines CAL27, SCC15, SCC9, and SCC25. Error bars represent the mean±S.D. from three independent experiments. (**P < 0.01). **b** After transfection of miR-103a-2-5p mimics in CAL27/LPS, the cell morphology transitioned from interstitial to epithelial (×200). **c** Western blot analysis showed that after transfection of miR-103a-2-5p mimics in CAL27/LPS, E-cadherin increased and vimentin and Twist1 decreased. **d** Immunofluorescence demonstrated an increase in E-cadherin and a decrease in vimentin after transfection of miR-103a-2-5p mimics in CAL27/LPS cells. **e** Transwell invasion and migration experiments confirmed that invasion and migration of tongue squamous cell carcinoma were reduced after transfection of miR-103a-2-5p mimics in CAL27/LPS cells. Error bars represent the mean±S.D. from three independent experiments. (**P < 0.01). **f** Western blotting revealed that transfection of miR-103a-2-5p in CAL27 and SCC15 cells overexpressing LTSCCAT rescued the expression of SMYD3, H3K4me3, and EMT-related biomarkers.

Previous research on miR-103a-2-5p was insufficient; thus, we further investigated the effect of miR-103a-2-5p on invasion and metastasis of TSCC and its mechanism. First, we tested the expression levels of miR-103a-2-5p in P.g-LPS-treated CAL27, SCC15, SCC9, and SCC25 cells and their untreated control cells and found that miR-103a-2-5p was upregulated in CAL27, SCC15, SCC9, and SCC25 cells (Fig. 6a). We upregulated the expression of miR-103a-2-5p by transfection of miR-103a-2-5p mimics in P.g-LPS-treated CAL27 cells and found that EMT was attenuated (Fig. 6b). The expression level of E-cadherin was upregulated and that of vimentin was downregulated (Fig. 6c, d). Moreover, the expression of Twist1, SMYD3, and H3K4me3 decreased (Fig. 6c). Transwell invasion and migration assays showed that cell invasion and migration were suppressed (Fig. 6e).

After overexpression of LTSCCAT in CAL27 and SCC15, western blot analysis showed a decrease in E-cadherin and an increase in vimentin, SMYD3, H3K4me3, and Twist1. Subsequently, miR-103a-2-5p mimics were transfected into CAL27 and SCC15 cells overexpressing LTSCCAT, which rescued the overexpression of LTSCCAT, upregulated E-cadherin, and downregulated vimentin, SMYD3, H3K4me3, and Twist1. Thus, LTSCCAT upregulates SMYD3 expression and promotes EMT by inhibiting the expression of miR-103a-2-5p (Fig. 6f).

### LncRNA LTSCCAT promotes the metastasis of xenograft tumors in vivo

**Fig. 7:**
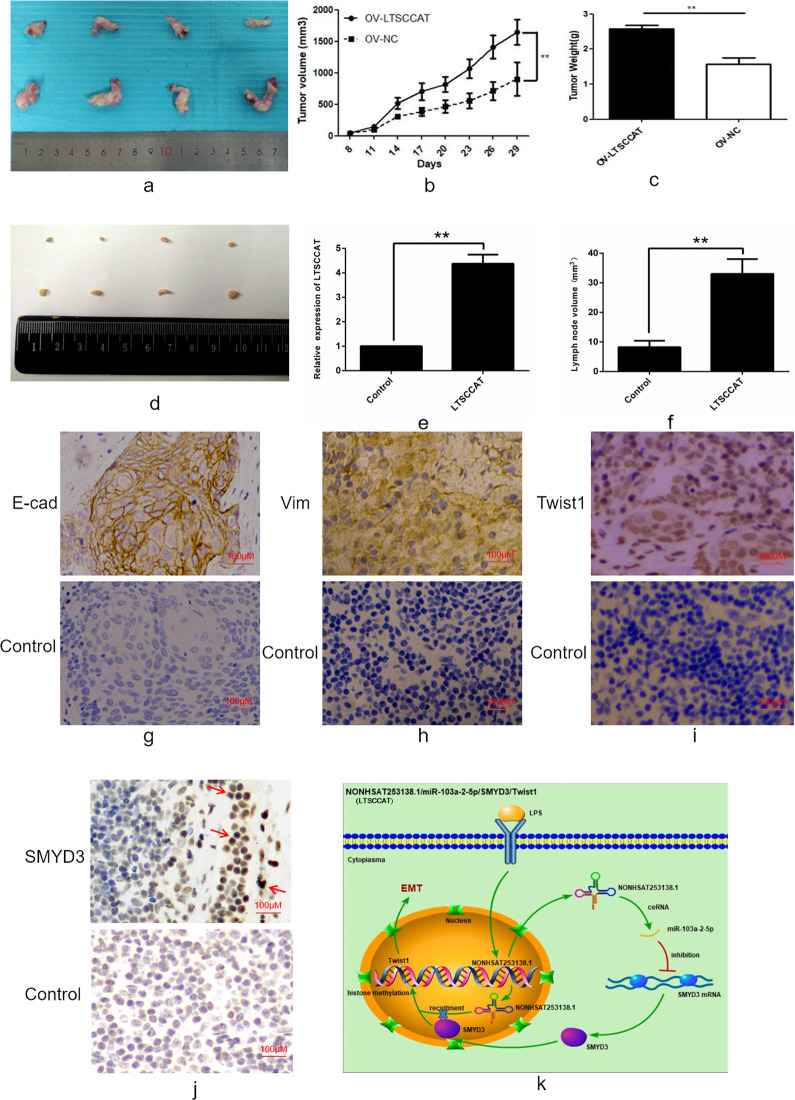
LTSCCAT has a significant impact on tumor development and metastasis in vivo. **a** Morphology of footpad transplanted tumors in mice inoculated with LTSCCAT overexpression CAL27 and negative control CAL27 cells. **b** Comparison of the size of transplanted tumors in overexpressed LTSCCAT group and control group over time. **c** The average weight of transplanted tumors in the overexpressing LTSCCAT group was greater than that in the control group. **d** The lateral axillary lymph node morphology of the inoculated LTSCCAT overexpression group and control group mice. **e** The expression level of LTSCCAT in the LTSCCAT overexpression group of transplanted tumors was higher than that in the control group. **f** The size of axillary lymph nodes in the LTSCCAT overexpression group was greater than that of the control group. **g** Immunohistochemistry showed the expression of E-cadherin in transplanted tumors. **h** Immunohistochemistry showed the expression of vimentin in transplanted tumors. **i** Immunohistochemistry showed the expression of Twist1 in transplanted tumors. **j** Immunohistochemistry showed the expression of SMYD3 in transplanted tumors. **k** Proposed model of the role of LTSCCAT in TSCC metastasis.

To further understand the effects of LTSCCAT on invasion and metastasis of TSCC in vivo, we next employed the subcutaneous tumor model of female nude mice to assess whether LTSCCAT can promote tumor metastasis. We generated LTSCCAT stably overexpressing CAL27 cells by lentiviral transfection. LTSCCAT overexpression CAL27 and negative control CAL27 cells were simultaneously injected into the right footpad of BALB/c female nude mice. We measured and recorded the growth of xenograft tumors in two groups of mice after inoculation (Fig. 7b). After the mice were killed, we collected footpad transplant tumors (Fig. 7a) and lateral axillary lymph nodes on the inoculation side. (Fig. 7d). The average weight of transplanted tumors in the overexpressing LTSCCAT group was greater than that in the control group (Fig. 7c). We extracted RNA from mouse footpad tumors, and QPCR detected the expression of LTSCCAT, which was higher in the overexpressed LTSCCAT group than in the control group (Fig. 7e). In addition, we also observed that the axillary lymph nodes in the overexpressed LTSCCAT group were slightly larger than the control group (Fig. 7f). We detected the expressions of E-cadherin, vimentin, Twist1, and SMYD3 in xenograft tissues by immunohistochemistry. The results showed that the expression of vimentin, Twist1, and SMYD3 was higher in the overexpressed LTSCCAT group than in the control group (Fig. 7h–j), whereas the expression of E-cadherin was lower in the overexpressed LTSCCAT group than in the control group (Fig. 7g).

## Discussion

TSCC is one of the most common malignant tumors among oral and maxillofacial tumors, often shows early lymph node metastasis and has a high metastatic rate. Furthermore, TSCC lymph node metastasis is associated with a poor prognosis and leads to decreased survival rates. Therefore, an in-depth study of the molecular mechanism of TSCC lymph node metastasis will provide new strategies for clinical prevention and treatment of TSCC metastasis. Periodontitis is a common chronic inflammation of the oral cavity, and its main pathogen, P.g, has been reported to be associated with various tumors, such as oral cancer, colorectal cancer, and pancreatic cancer^29–31^. Studies have shown that the colonization level of P.g in oral squamous cell carcinoma is significantly higher than that of adjacent tissues, and it can enhance the invasion and migration of gingival epithelial cells^32–34^. For instance, P.g can initiate a mesenchymal-like transition through ZEB1 in gingival epithelial cells^35^. However, the molecular mechanisms by which periodontitis and P.g promote metastasis of TSCC are unclear. LncRNAs are one of the common regulatory mechanisms of epigenetic modification^36^. Many studies have indicated that LncRNA expression is dysregulated during tumorigenesis and progression. However, studies on the molecular mechanism of LncRNAs in periodontitis and P.g promotion of TSCC invasion and metastasis are limited, and thus, analysis of the detailed molecular mechanism is urgently needed.

Epigenetic regulation has an important role in gene expression, including pre-transcriptional regulation, regulation of transcription, post-transcriptional regulation, translational regulation, and post-translational regulation. Regulatory mechanisms include chromatin remodeling, DNA methylation, histone modification, X-chromosome inactivation, and noncoding RNA regulation. Among them, LncRNAs can participate in the regulation of the expression of target genes at multiple levels. LncRNAs have a relatively long-nucleotide chain with a specific and complex secondary spatial structure inside the molecule, which can provide multiple sites for binding to proteins and can also specifically bind DNA or RNA through the principle of base complementary pairing, thus producing complex and precise regulation of gene expression. This regulatory mechanism depends on the cellular location of the lncRNA^37^. In this study, we revealed that LncRNA LTSCCAT is highly expressed in TSCC cells induced by P.g-derived LPS through LncRNA sequencing. Highly expressed LTSCCAT promoted invasion and metastasis of TSCC and led to a poor prognosis, suggesting that LTSCCAT expression functioned as an independent prognostic factor in TSCC patients. LTSCCAT had higher expression in TSCC patients with periodontitis than in TSCC patients without periodontitis, indicating that periodontitis can promote invasion and metastasis of TSCC by promoting LTSCCAT expression.

During EMT, epithelial cells transform into mesenchymal cells, and this process is involved in embryonic development, chronic inflammation, and cancer metastasis^38^. This cell differentiation and behavioral switching are mediated by key transcription factors such as Snail, ZEB, Twist1^39^. Through the process of EMT, epithelial cells lose the epithelial phenotype, including cell polarity and attachment to the basement membrane, and gain a more interstitial phenotype involving migration and invasion. We observed that TSCC cells underwent EMT after P.g-LPS treatment and exhibited elevated LTSCCAT. Downregulation of LTSCCAT in P.g-LPS-treated TSCC cells underwent MET, whereas LTSCCAT upregulation in untreated TSCC cells was observed during EMT. Upregulation of LTSCCAT in TSCC cells led to increased levels of the EMT-related transcription factor Twist1. Thus, the level of LTSCCAT may be a switch to regulate EMT/MET in TSCC, affecting its progression and prognosis, whereas decreasing LTSCCAT expression in TSCC may promote MET and result in a better prognosis.

ISH and nucleoplasm separation experiments verified that LTSCCAT is mostly expressed in the cytoplasm. LTSCCAT in the cytoplasm may act as a ceRNA in binding to miRNA at the post-transcriptional level. We then used bioinformatics analysis to predict LTSCCAT’s potentially competitively bound miRNAs and identified miR-103a-2-5p, which regulates invasion and metastasis of TSCC. To further investigate the ceRNA mechanism of LTSCCAT, we confirmed that LTSCCAT binds to miR-103a-2-5p at the 623–645 and 1077–1103 regions by dual-luciferase reporter gene assays. We further investigated the functional mechanism of miR-103a-2-5p and confirmed the presence of the binding site of miR-103a-2-5p in the 3′UTR of SMYD3 by online software prediction and dual-luciferase reporter gene assays.

Histone modification is a post-translational modification of nucleosome core histones by related enzymes; these processes include methylation, phosphorylation, acetylation, ubiquitination, adenylation, and ADP ribosylation. Methylation of histones refers to varying degrees of methylation occurring at different sites of the N-arginine or lysine residues of the H3 and H4 histones, catalyzed by histone methyltransferases containing SET domains^40,41^. Histone methylation can affect the affinity of histones and DNA duplexes to alter the state of chromatin, resulting in loose or compact chromatin, and can also affect the binding of transcription factors to the promoter sequence of structural gene DNA. Trimethyl-H3-K4 (H3K4me3) is enriched in active transcriptional genes and can recruit factors involved in the regulation of gene transcription^42–44^. In our study, we performed chromatin immunoprecipitation with the SMYD3 antibody, purified the precipitated DNA and detected it with real-time quantitative PCR, confirming that SMYD3 directly binds to the Twist1 promoter and promotes Twist1 transcription. In addition, the ChIP experiment revealed that this is related to the level of trimethylation of H3K4.

The results of the RF classifier and SVW classifier showed that LTSCCAT likely directly interacts with SMYD3. However, RIP assays revealed that LTSCCAT could not interact with SMYD3 in TSCC cells, indicating that LTSCCAT cannot directly bind to SMYD3.

In conclusion, we showed that LTSCCAT acts as an oncogene, is highly expressed in TSCC tissues from TSCC patients with periodontitis compared with those without periodontitis, and exerts a significant role in promoting the metastasis of TSCC by promoting the occurrence of EMT. Furthermore, for the first time, our study investigated LncRNA LTSCCAT competes with miR-103a-2-5p and upregulates the SMYD3/Twist1 signaling pathway to promote TSCC migration, and we showed that LTSCCAT cannot directly bind to SMYD3 (Fig. 7e). According to our clinical evidence, this LncRNA can act as an independent prognostic factor for TSCC patients.

## Materials and methods

### Cell culture and stimulation

The CAL27, SCC9, SCC25, and SCC15 cell lines were obtained from ATCC (Manassas, VA, USA) and were authenticated recently. The test for mycoplasma contamination was performed and there was no mycoplasma contamination. CAL27, SCC9, SCC25, and SCC15 cells were respectively grown in Dulbecco’s Modified Eagle Medium (DMEM) and DMEM/F-12 medium (Gibco) supplemented with 10% FBS (Biological Industries, Beit- Haemek Israel) and 1% antibiotics (Gibco, 100 U/ml penicillin and 100 μg/ml streptomycin) and cultured in a humidified incubator at 37 °C and 5% CO_2_. P.g-LPS (standard) (Invitrogen, USA) was used to treat CAL27, SCC15 cell lines at 20 µg/ml for 6 days.

### LncRNA sequencing and bioinformatics analysis

Analysis using human LncRNA expression sequencing provided by BGI (Shenzhen, China) was performed as previously described with P.g-LPS treated CAL27 and untreated CAL27 cells.

The RegRNA 2.0 website was constructed and maintained by scientists at the National Chiao Tung University in Taiwan and was used to predict the presence of LTSCCAT binding to miR-103a-2-5p. The website is regrna2.mbc.nctu.edu.tw^28^. The online software prediction results also indicated that the 3′-UTR of SMYD3 has a binding site to miR-103a-2-5p.

### Patients and tissue samples

A total of 118 specimens of TSCC were obtained from the Department of Oral and Maxillofacial Surgery, Sun Yat-sen Memorial Hospital, and were divided into two groups with or without periodontitis. The patients with incomplete follow-up information were excluded. All of these specimens (clinical stage III or IV) were enrolled from 2012 to 2017 and were stored in liquid nitrogen before analyses. Informed consent was obtained from all the patients who provided the samples, and the study was approved by the Ethics Committee of Sun Yat-sen Cancer Center.

### Nuclear-plasma fractionation

First, 10^7^ cells were digested and washed twice with precooled PBS, and PBS was removed after centrifugation. The pellet was resuspended in a centrifuge tube by adding 1 ml of RNase-free PBS, 1 ml of buffer C1 (1.28 M sucrose, 40 mM Tris, pH 7.5, 20 mM MgCl_2_, 4% Triton X-100) and 3 ml of diethyl pyrocarbonate water. The samples were placed on ice for 15 minutes and centrifuged at 3000 rpm for 15 minutes. After centrifugation, the supernatant was the cytoplasm, and precipitate was the nucleus.

### RACE assay

The total RNA of CAL27 was extracted and reverse transcribed into cDNA, and then, PCR amplification was carried out using the designed primers, and the PCR product was purified and sequenced. The sequencing results showed a set of peaks that required TA clone sequencing. The primers (Supplementary Table S1) were designed to further verify the results obtained by the RACE experiment. After PCR amplification, 1% agarose gel electrophoresis was performed to amplify the target fragment, which was consistent with the expected length.

### Cell transfection

The specific siRNAs targeting LTSCCAT and SMYD3 were obtained from GenePharma (Shanghai, China). The indicated cells were transfected with 75 nM siRNA using Lipofectamine RNAiMAX (Invitrogen, USA) according to the manufacturer’s protocol. The targeting sequences are listed in the supplementary file.

### Transwell invasion and migration assay

The Transwell invasion assay used a 24-well transwell chamber with a pore size of 8 µl. After the treated cells were digested, an equal amount of complete medium was added to neutralize the trypsin, and after centrifugation, the cells were resuspended in serum-free medium. After the cells were quantified, they were added to the transwell upper chamber at 8 × 10^4^ cells/well. Each upper chamber required 200 µl of serum-free medium, and 600 µl of complete medium was added to the lower chamber and then cultured in a 37 °C incubator for 10–20 hours (different cell at different times). After the incubation time, the chamber was removed and placed in a 24-well plate containing 600 µl of 4% paraformaldehyde for 30 minutes. It was then dyed for 30 minutes in the crystal violet solution. The excess dye solution was washed off, the samples were dried and observed under a microscope and imaging and quantification were performed.

### Quantitative real-time PCR

We extracted total RNA from tissue samples and cells and synthesized the first strand of cDNA based on total RNA using the RevertAid First Strand cDNA Synthesis Kit (Fermentas) following the manufacturer’s instructions. Real-time PCR was performed with SYBR® Premix Ex Taq™ II (Tli RNaseH Plus) (TaKaRa), with GAPDH as an endogenous control. We determined the expression levels of the relative genes by the 2−ΔΔCt method, and the primer sequences are shown in the supplementary file (Supplementary Table S1).

### Western blot analysis

Total protein was extracted from cells using RIPA and Protease Inhibitor Cocktail to detect the expression levels of related proteins in cells. The protein concentration was determined using a BCA assay (Beyotime, China) according to the manufacturer’s information. A total of 30 µg of total protein was applied to a 10% sodium dodecyl sulphate–polyacrylamide gel electrophoresis and then transferred to a polyvinylidene fluoride membrane. The membrane was then blocked in 5% skim milk powder for 1 h, washed with TBST and incubated in primary and secondary antibodies, and finally imaging with an ECL chemiluminescence kit (Beyotime, China).

The primary antibodies used in the experiments included anti-E-cadherin (Santa Cruz, USA, sc-21791), anti-TWIST1 (Santa Cruz, USA,sc-81417), anti-vimentin (Santa Cruz, USA, sc-66001), anti-GAPDH (Ray Antibody, Beijing, China, RM2002) anti-SMYD3 (Abcam, USA, ab-85277), and anti-H3K4me3 (Cell Signaling Technology, USA, 9751 s), goat anti-mouse IgG (Proteintech, China, SA00001-1) and goat anti-rabbit IgG secondary antibodies (Proteintech, China, SA00001-2).

### Immunofluorescence staining

The cells were cultured on coverslips in six-well plates and then fixed in 4% paraformaldehyde. The cells were incubated with primary antibodies against E-cadherin and vimentin (Santa Cruz, CA, USA) and then incubated with secondary antibodies. The slides were examined using a fluorescence microscope (Olympus). Green fluorescence showed localization of E-cadherin and red fluorescence showed localization of vimentin. The nuclei were counterstained with 4’,6-diamidino-2-phenylindol.

### Stable LTSCCAT overexpression cell lines

Lentiviral vectors overexpressing LTSCCAT or a negative control vector were obtained from GenePharma (Shanghai, China). Then, CAL27 cells were infected with lentivirus vector overexpressing LTSCCAT and negative control vector and were selected with 2 μg/ml puromycin for 2 weeks.

### Immunohistochemistry

Immunohistochemistry was performed on paraffin-embedded mouse transplanted tumor tissue sections. The tissue sections were incubated with SMYD3, E-cad, vimentin and Twist1 primary antibody at 4 °C overnight. The secondary antibody was incubated for 30 minutes at room temperature. The tissue sections were observed under a Zeiss AX10-Imager A1 microscope (Carl Zeiss, Thornwood, NY, USA) and processed with AxioVision 4.7 microscopy software (Carl Zeiss, Thornwood, NY, USA).

### ISH

ISH was performed to detect the expression and location of LTSCCAT in TSCC tissue and normal adjacent tissue specimens. In brief, paraffin slides were dewaxed in xylene and washed with ethanol. Exposure of the nucleic acid to 0.5% Triton X-100 and RNase-free protein K enhances the probe hybridization to the target gene. The specimen was then incubated with specific probes for 16 hours at 42 °C. After the sample was incubated for 30 minutes using a 1% Roche blocking solution, the specimen was incubated with horseradish peroxidase-conjugated anti-DIG antibody overnight at 4 °C. The nuclei were counterstained with hematoxylin. Finally, we captured and analyzed the images.

### Chromatin immunoprecipitation assay

ChIP assays were performed using the EZ-Magna chip A/G Chromatin Immunoprecipitation Kit (Millipore, Massachusetts, USA) following the manufacturer’s instructions. First, 107 cells were collected per reaction and fixed in 1% formaldehyde for 10 minutes at room temperature. After the cells were collected, they were resuspended with cell lysis buffer and nuclear lysis buffer, and then, isolated chromatin was sonicated to shear DNA. Antibodies targeting SMYD3, H3K4me3, and IgG were used for each assay. Purified DNA was used to evaluate the enrichment of SMYD3 and H3K4me3 by qRT-PCR. The primers involved in these assays are listed in the supplementary file (Supplementary Table S1).

### RIP assays

RIP experiments were performed using the Magna RIP RNA-Binding Protein Immunoprecipitation Kit (Millipore, Billerica, MA, USA) according to the manufacturer’s instructions. RIP experiments were performed using the Magna RIP RNA-Binding Protein Immunoprecipitation Kit (Millipore, Billerica, MA, USA) according to the manufacturer’s instructions. The SMYD3 antibody for RIP assays was from Abcam, USA, and the control IgG antibody was from Millipore. The coprecipitated RNAs were detected by qRT-PCR. The total RNAs were the input controls.

### Tumorigenicity assays in nude mice

The 4–6 weeks old female BALB/c-nu mice were purchased from the Animal Center of Sun Yat-sen University. A total of eight mice were selected and randomly divided into two groups by tossing coins: the NC group and ov-LTSCCAT group, and four mice were in each group. The investigators were blinded to the group allocation during the experiment and when assessing the outcome. CAL27 cells stably transfected with lentivirus were trypsinized, and 5 × 10^6^ cells were counted per mouse. The counted cells were injected into the right footpad of nude mice. The Animals which was failure in tumor xenografts were excluded. The maximum diameter A and minimum diameter B of tumors of tumor-forming mice were measured with vernier calipers, and tumor volume changes of mice were calculated and recorded. Mouse tumor volume is approximately equal to A × B × B × 0.5. On the 30th day, the experiment was terminated, the mice were sacrificed, and the tumors of the mice and the axillary lymph nodes of the inoculation were isolated. Weigh and record the tumor weight of two groups of mice and measure the lymph node volume (the calculation method is the same as the tumor volume). Tumors and lymph node tissues were sent for sectioning for HE staining, and RNA was extracted to detect gene expression.

### Dual-luciferase reporter assay

According to the RegRNA 2.0 prediction, we designed mutants for each potential miR-103a-2-5p binding site in the LTSCCAT sequence and cloned the mutant and wild-type LTSCCAT into the luciferase plasmid. Next, we cotransfected these plasmids with miR-103a-2-5p into HEK293T cells. Similarly, we cloned wild-type SMYD3 and mutant SMYD3 into a luciferase plasmid and cotransfected them with mimics and inhibitors of miR-103a-2-5p. After 48 hours of incubation, luciferase activity was measured and analyzed using a dual-luciferase reporter assay system (Promega, USA). A reporter plasmid containing Renilla luciferase was used as a standard reference to calculate the ratio of firefly fluorescence to Renilla fluorescence.

### Study approval

Patient study was performed in accordance with the Declaration of Helsinki. The Sun Yat-sen University Committee provided the Ethics approval. The patients of TSCC were identified and provided the informed consent under 2015144 from Sun Yat-sen Memorial Hospital. The Animal Ethics Committee of Sun Yat-sen University formally approved the animal experiments.

### Statistical analysis

All statistical analyses were performed using SPSS 20.0 software (IBM, SPSS, Chicago, IL, USA) and GraphPad Prism 6. Comparisons between the two groups were performed using a two-tailed Student’s *t* test or Wilcoxon test, depending on the type of data. All experiments were performed at least three times, and the results of the experiment are expressed as the mean ± SD. Kaplan–Meier survival curves were plotted, and a log-rank test was used. A *P* value of <0.05 was considered significant.

## Supplementary information

Figure S1

table S1

Supplementary figure and table legends

## Data Availability

Data have been deposited in the Sequence Read Archive (SRA) DataSets (https://www.ncbi.nlm.nih.gov/home/submit) under the following accession number: PRJNA610015.
