# Supplementary figures and images for: LncRNA LTSCCAT promotes tongue squamous cell carcinoma metastasis via targeting the miR-103a-2-5p/SMYD3/TWIST1 axis
Source: Cell Death Dis. 2021 Feb 1;12(2):144. doi: 10.1038/s41419-021-03415-2 (PMC7862618; doi:10.1038/s41419-021-03415-2)

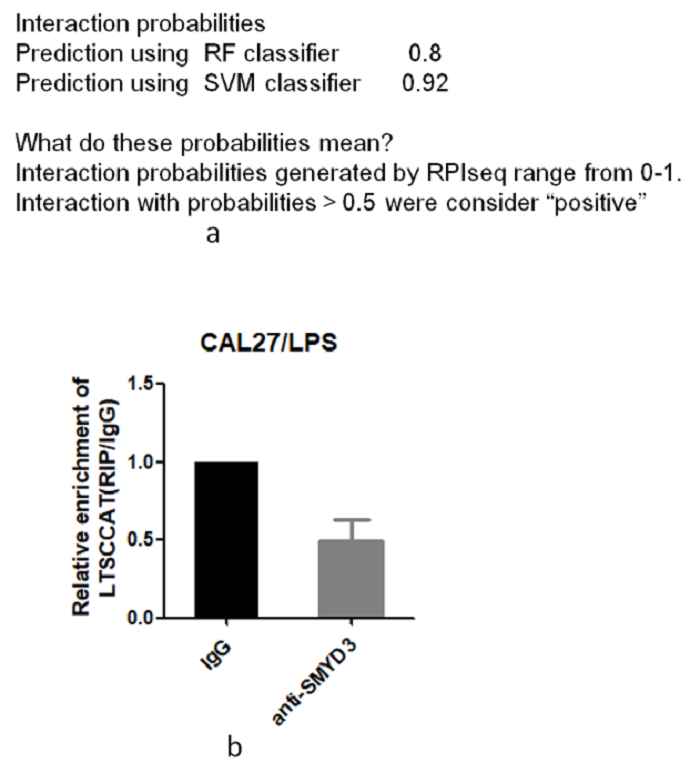

Supplement: Supplementary file 1 — Figure S1 [file 41419_2021_3415_MOESM1_ESM.tif]
