# Supplementary material for: LncRNA LTSCCAT promotes tongue squamous cell carcinoma metastasis via targeting the miR-103a-2-5p/SMYD3/TWIST1 axis
Source: Cell Death Dis. 2021 Feb 1;12(2):144. doi: 10.1038/s41419-021-03415-2 (PMC7862618; doi:10.1038/s41419-021-03415-2)
Supplement: Supplementary file 2 — table S1 [file 41419_2021_3415_MOESM2_ESM.docx]

| Primers and siRNAs | Forward | Reverse |
| --- | --- | --- |
| LTSCCAT | CGCTCATGGGCTTCTCAGTA | AGCTCCACACCCTGAGGTTT |
| Has-miR-103a-2-5p MIMAT0009196 | AGCUUCUUUACAGUGCUGCCUUG |  |
| Has-miR-103a-2-5p mimic | AGCUUCUUUACAGUGCUGCCUUG | CAAGGCAGCACUGUAAAGAAGCU |
| Has-miR-103a-2-5p inhibitor | CAAGGCAGCACUGUAAAGAAGCU |  |
| SMYD3 | GTTCGATTGTGTTCAATGGGCCCCAC | TCCTCACTGGTCATCAGCATATC |
| LTSCCAT 623-645 mutant | GGTGCTCGCCCACAATAGAAGTTCACTCTTCGGTGGAAGGAAACCCAGTCCATG | GAACTTCTATTGTGGGCGAGCACCTTCCGAGGCGTAGTCCTTTGGATGTTGGGG |
| LTSCCAT 1077-1103 mutant | GCCTTGGAGCCACCATCCATCTTTTGCAATGCCATACTTGTTTCCTTTTTGAAA | AAAAGATGGATGGTGGCTCCAAGGCGACTTCCTTTCTGGTTCACCAACTTTGCT |
| SMYD3 mutant | TCATTAGTTGTAGCACCATCCGATTATAATAAATTCAAAACATTTGGTTG | TAATCGGATGGTGCTACAACTAATGATTCTTGTGGTTTGCAAACCATGTC |
| Twist1 promoter1 | GGTAAGGGATCAACTCTGCAA | CGACTGAAGGGTTCAGCTTT |
| Twist1 promoter2 | TTTCCTTCCACCGAAGAGTG | CCCTCCTTTCCCCTGTACAA |
| Twist1 promoter3 | AGTCTCCTCCGACCGCTTC | GTCCTCCCAAACCATTCAAA |
| Twist1 promoter4 | TTGGGAGGACGAATTGTTAGA | ACGTGAGGAGGAGGGACTTT |
| LTSCCAT siRNA1 | AGCATTTCTGTCTGTAAGT |  |
| LTSCCAT siRNA2 | CGACTTGACCTCCACTTAA |  |
| LTSCCAT siRNA3 | GGACAAATTGCAGAGTTGA |  |
| SMYD3 siRNA1 | GCAAGUAUGGAAGGAAGUU |  |
| SMYD3 siRNA2 | AGUAUCUCUUUGCUCAAUCAA |  |
| SMYD3 siRNA3 | CAAGUAUGGAAGGAAGUUCAA |  |
| 5’RACE |  |  |
| CTG1064-2 R1 | ACTGGGTCGTTGTAGAGGGGAAAG |  |
| CTG1064-2 R4 | ACTCCAAGGGGTTCGTCTACCTGA |  |
| CTG1064-2 R5 | TGCTGCCCCCAAACTTTCCGCCTG |  |
| CTG1064-2 YZF1 | AATCTTGCTCAGCTTGTCCGAGGG |  |
| CTG1064-2 YZF2 | CGCACGTTGGCCATGACCCGCTGC |  |
| 3’RACE |  |  |
| CTG1064-1 F1 | AACCCTAGCGCAGCCCAGGA |  |
| CTG1064-1 R1 | TCGAGATTTCTGCAGCCACG |  |
| CTG1064-1 F2 | GTGACAGCAGCAATGGCAAC |  |
| CTG1064-1 R2 |  |  |
| CTG1064 P1 | CCGTACTGAGAAGCCCATGA |  |
| CTG1064-1 F3 | TTTGGGAGAAAGTGAGGGCGAGTGT |  |
| CTG1064-1 F5 | CCTGTTTAGTGAGCCGCTGCCTTA |  |
| CTG1064-1 F6 | TTTGGGAGAAGTGAGGGCGAGTGT |  |
| CTG1064-1 F7 | CCTGTTTAGTGAGCCGCTGCCTTA |  |

**Table S1 The primers and siRNAs involved in these assays of this study.**
