# Supplementary material for: LncRNA LTSCCAT promotes tongue squamous cell carcinoma metastasis via targeting the miR-103a-2-5p/SMYD3/TWIST1 axis
Source: Cell Death Dis. 2021 Feb 1;12(2):144. doi: 10.1038/s41419-021-03415-2 (PMC7862618; doi:10.1038/s41419-021-03415-2)
Supplement: Supplementary file 3 — Supplementary figure and table legends [file 41419_2021_3415_MOESM3_ESM.docx]

**Figure S1 LTSCCAT cannot directly binds to the SMYD3 protein.**

**a** The results of RF classifier and SVW classifier analysis showed that LTSCCAT probably directly interacts with SMYD3. **b** RIP assays revealed that LTSCCAT could not interact with SMYD3 in TSCC cells.

**Table S1 The primers and siRNAs involved in these assays of this study.**
